# Improved Repopulation Efficacy of Decellularized Small Diameter Vascular Grafts Utilizing the Cord Blood Platelet Lysate

**DOI:** 10.3390/bioengineering8090118

**Published:** 2021-08-27

**Authors:** Panagiotis Mallis, Dimitrios P. Sokolis, Michalis Katsimpoulas, Alkiviadis Kostakis, Catherine Stavropoulos-Giokas, Efstathios Michalopoulos

**Affiliations:** 1 Hellenic Cord Blood Bank, Biomedical Research Foundation Academy of Athens, 4 Soranou Ephessiou Street, 115 27 Athens, Greece; cstavrop@bioacademy.gr (C.S.-G.); smichal@bioacademy.gr (E.M.); 2 Laboratory of Biomechanics, Center for Experimental Surgery, Biomedical Research Foundation Academy of Athens, 4 Soranou Ephessiou Street, 115 27 Athens, Greece; dsokolis@bioacademy.gr; 3 Center of Experimental Surgery and Translational Research, Biomedical Research Foundation Academy of Athens, 4 Soranou Ephessiou Street, 115 27 Athens, Greece; mkatsiboulas@bioacademy.gr (M.K.); akostakis@bioacademy.gr (A.K.)

**Keywords:** decellularization, human umbilical arteries, mesenchymal stromal cells, repopulation, Ki67, MAP kinase

## Abstract

Background: The development of functional bioengineered small-diameter vascular grafts (SDVGs), represents a major challenge of tissue engineering. This study aimed to evaluate the repopulation efficacy of biological vessels, utilizing the cord blood platelet lysate (CBPL). Methods: Human umbilical arteries (hUAs, *n* = 10) were submitted to decellularization. Then, an evaluation of decellularized hUAs, involving histological, biochemical and biomechanical analysis, was performed. Wharton’s Jelly (WJ) Mesenchymal Stromal Cells (MSCs) were isolated and characterized for their properties. Then, WJ-MSCs (1.5 × 10^6^ cells) were seeded on decellularized hUAs (*n* = 5) and cultivated with (Group A) or without the presence of the CBPL, (Group B) for 30 days. Histological analysis involving immunohistochemistry (against Ki67, for determination of cell proliferation) and indirect immunofluorescence (against activated MAP kinase, additional marker for cell growth and proliferation) was performed. Results: The decellularized hUAs retained their initial vessel’s properties, in terms of key-specific proteins, the biochemical and biomechanical characteristics were preserved. The evaluation of the repopulation process indicated a more uniform distribution of WJ-MSCs in group A compared to group B. The repopulated vascular grafts of group B were characterized by greater Ki67 and MAP kinase expression compared to group A. Conclusion: The results of this study indicated that the CBPL may improve the repopulation efficacy, thus bringing the biological SDVGs one step closer to clinical application.

## 1. Introduction

The development of functional small-diameter vascular grafts (SDVGs), which can effectively be used in cardiovascular applications, remains a great challenge. Cardiovascular disease (CVD) represents a wide group of disorders, including peripheral arterial disease (PAD), coronary artery disease (CAD), cerebrovascular disease and rheumatic heart disease [1,2,3,4]. Changes in daily routine by adopting different behavioral habits, such as dietary change, smoking limitation and body exercise, may reduce the incidence of CVD [5,6,7,8,9]. However, CVD is one of the leading causes of death worldwide, estimating that more than 17 million people are suffering from some form of CVD [7]. In addition, more than 500,000 bypass surgeries are performed on CVD patients in the USA each year [10,11]. Therefore, the appropriate administration of CVD may result in a reduction in the corresponding global burden of death.

Different CVD cases require different therapeutic approaches, from medications’ initiation to vascular graft replacement. Indeed, in the case of PAD and CAD, the utilization of vascular graft substitutes represents the most effective approach [12,13,14]. Currently, the gold standard approach for CAD is the replacement of the occluded vessel with another SDVG substitute [15,16]. Most times, secondary vascular grafts such as the saphenous vein and the mammary and radial arteries are preferred [16]. However, less than 50% of CVD patients are characterized by an adequate vascular network. It is known that CVD can cause significant hemodynamic differences throughout the whole patient’s vascular network. In addition, accumulated atherosclerotic plaque in combination with stiffer blood vessels are common manifestations in CVD patients. Therefore, the utilization of suitable autologous secondary vascular grafts (e.g., saphenous vein), in order to be used for bypass grafting in those patients, is a demanding task [2,16]. As an alternative to autologous vessels, synthetic SDVGs can represent an important candidate [16]. The most used materials for vascular grafts fabrication are Dacron and ePTFE. Although synthetic vascular grafts made from Dacron and ePTFE have been used with promising results, in large diameter vessel replacement (e.g., aorta), their application as SDVGs is followed by severe adverse reactions by the host [17,18]. These adverse reactions may include the extended immune response against the synthetic vessel, platelet aggregation followed by clot formation, calcification development at the anastomoses sites, which further promote the graft rejection [17,18]. Moreover, the above-described adverse reactions may be deleterious for the patients and can even be life-threatening. Additionally, the use of synthetic SDVGs in pediatric patients is still not a favorable approach, unless there is no other alternative option [2,16]. In the past, also, cross-linked (formalin-fixed) vascular grafts of animal origin have been evaluated as vessel substitutes [19,20]. This type of grafts was characterized by totally different mechanical properties compared to the resident vessels, which could result in neointima formation and graft occlusion, due to compliance mismatch [16]. Moreover, formalin-fixed grafts have a limited in vivo remodeling ability; therefore, their potential application in CAD is reduced.

In the context of suitable SDVG development, the utilization of advanced tissue engineering approaches may guarantee an important solution to address the above issues [2]. In this way, the application of the decellularization method to produce SDVGs has been evaluated by various research groups worldwide [21,22,23]. Considering the above data, the potential use of decellularized human umbilical arteries (hUAs) has been proposed to be used as suitable SDVGs for CAD applications [21,22,23].

More specifically, the human umbilical cord (hUC) consists of two arteries and one vein [24,25,26]. The average length of the human umbilical cord can reach 50–60 cm [24,25]. The hUAs are responsible for the transportation of the non-oxygenated blood from the fetus to the mother. It has been calculated that more than 40 L of blood are transported through the hUAs. Histological analysis of the hUAs has revealed the presence of three layers in the vascular wall, the tunica intima (TI), media (TM) and adventitia (TA) [24,25]. Each layer is characterized by the presence of different cell populations, including the endothelial cells (in TI), the vascular smooth muscle cells (in TM) and the perivascular cells (in TA) [24,25,26]. In addition, hUAs are characterized by a lumen diameter of 2–6 mm and can be non-invasively isolated after gestation. Therefore, hUAs may represent an important candidate for the development of SDVGs.

To date, several research groups have evaluated the decellularized human umbilical vessels as vascular graft substitutes [21,22,23]. Decellularization aims to eliminate the vessel’s cell populations, maintaining in parallel the integrity of the extracellular matrix (ECM) [26,27,28]. However, the repopulation of the vascular graft with host cells must be performed in order to gain its original functionality [29]. Most times, the proper repopulation (with the desired cellular populations, e.g., endothelial cells and vascular smooth muscle cells) of the decellularized vascular grafts is performed using vessel bioreactors under defined conditions [29]. However, the repopulation efficacy is still low; therefore, an improvement of the whole procedure may be considered. In such a way, the use of CBPL may be applied as an additive to the repopulation process in order to improve cell adhesion. From advanced proteomic analysis, it has been shown that the CBPL contains a significant amount of growth factors including the platelet-derived growth factor (PDGF), transforming growth factor-β1 (TGF-β1), fibroblast growth factor (FGF), cytokines such as tumor necrosis factor-a (TNF-α), interleukin (IL)-1, IL-3, IL-6 and matrix metalloproteases [30,31,32,33]. The concentration and the combination of the above proteins have been shown to present tissue remodeling properties and also favor cell adhesion, proliferation and differentiation [34]. Currently, CBPL has been used widely in personalized regenerative medicine applications including wound and burn healing, and the regeneration of long-term skin ulcers in diabetic patients, while the standardization criteria for its proper production have also been described and published [35,36,37]. Therefore, the utilization of CBPL may improve the repopulation efficacy of the decellularized hUAs.

This study aimed to evaluate the impact of CBPL as a culture mediator for the improvement of SDVGs’ repopulation. For this purpose, decellularized hUAs will be used as a potential scaffold for the repopulation experiments. MSCs derived from hUCs were seeded onto the decellularized hUAs and cultured in the presence of a cultivation medium containing CBPL. To evaluate the repopulation process, histological and biochemical analyses of the recellularized vessels were performed. The results of this study may deepen knowledge on efficient vascular graft development.

## 2. Materials and Methods

### 2.1. Isolation of hUAs

HUAs (*n* = 60, l = 4 cm) were isolated from the hUCs that were delivered to the Hellenic Cord Blood Bank (HCBB). All hUC samples were collected from end-term normal or caesarian deliveries (gestational ages 38–40 weeks) by experienced midwives. The informed consent for the enrolment of the current study was signed by the mothers before the gestation. The informed consent of the current study was in accordance with the ethical standards of the Greek National Ethical Committee and fulfilled the criteria of the Helsinki Declaration. The overall study was approved by the Bioethics Committee of BRFAA (No 2843, 7 October 2020). After the delivery to the HCBB, the hUCs were kept in Phosphate Buffer Saline 1× (PBS 1x, Gibco, Life Technologies, Grand Island, NE, USA) supplemented with 10 U/mL Penicillin and 10 μg/mL Streptomycin (Gibco, Life Technologies, Grand Island, NE, USA). The hUAs’ isolation was performed within 24 h after the hUCs delivery. Briefly, the hUCs were rinsed in PBS 1x to remove the excess blood and blood clots. Then, isolation of intact hUAs was performed with the use of sterile surgical instruments. HUAs with occluded lumen were not used for the current experimental procedure and were discarded. Finally, each hUA was separated into two segments of 2 cm. The one-segment (l = 2 cm) was served as native hUA, whereas the other segment (l = 2 cm) was submitted to decellularization.

### 2.2. Decellularization of hUAs

The hUAs were decellularized based on an already published protocol from our research team [38]. Briefly, hUAs (*n* = 60, l = 4 cm) were placed in the first decellularization solution, which consisted of 8 mM CHAPS, 1 M NaCl and 25 mM EDTA in PBS 1x (Sigma-Aldrich, Darmstadt, Germany) for 22 h at room temperature (RT). Then, the hUAs were briefly washed in PBS 1x to remove the excess of the initial decellularization solution. After this step, the hUAs were placed in the second decellularization solution, which consisted of 1.8 mM SDS, 1 M NaCl and 25 mM EDTA in PBS 1x, (Sigma-Aldrich, Darmstadt, Germany) for another 22 h at RT, followed by a brief wash in PBS 1x. Finally, the hUAs were incubated in α-Minimum Essentials Medium (α-MEM, Sigma-Aldrich, Darmstadt, Germany) and supplemented with 40% Fetal Bovine Serum (FBS, Sigma-Aldrich, Darmstadt, Germany) for 48 h at 37 °C. All steps were performed under rotational and continuous agitation.

### 2.3. Histological Analysis of hUAs

The evaluation of the elimination of cellular populations and the ECM preservation in decellularized hUAs was performed with the histological analysis. Specifically, native (*n* = 5, l = 2 cm) and decellularized (*n* = 5, l = 2 cm) hUAs were fixed in 10% *v*/*v* neutral formalin buffer (Sigma-Aldrich, Darmstadt, Germany), dehydrated, paraffin-embedded and sectioned at 5 μm. Hematoxylin and Eosin (H&E, Sigma-Aldrich, Darmstadt, Germany), Orcein Stain (OS, Sigma-Aldrich, Darmstadt, Germany), Masson’s Trichrome (MT, Sigma-Aldrich, Darmstadt, Germany) and Toluidine Blue (TB, Sigma-Aldrich, Darmstadt, Germany) were used for the evaluation of cellular/nuclear materials, elastin, collagen and sulphated glycosaminoglycans (sGAGs), respectively. Images were acquired with a Leica DM L2 light microscope (Leica Microsystems, Weltzar, Germany) and processed with ImageJ 1.46 r (Wane Rasband, National Institutes of Health, Bethesda, MD, USA).

### 2.4. Scanning Electron Microscopy Analysis of hUAs

Scanning electron microscopy (SEM) analysis was performed to further evaluate the ultrastructure of the hUAs. Specifically, hUA segments obtained from native (*n* = 5, l = 8 mm) and decellularized (*n* = 5, l = 8 mm). Then, all samples were initially fixed with 1% *v*/*v* glutaraldehyde solution (Sigma-Aldrich, Darmstadt, Germany). Briefly, rinses with distilled water were performed 3 times. Then, dehydration of segments was performed using 70% *v*/*v*, 80% *v*/*v*, 95% *v*/*v* aqueous ethanol and absolute ethanol for 20 min each. Dehydrated hUA segments were placed in hexamethyldisilazane solution (Sigma-Aldrich, Darmstadt, Germany) for 10 min, air-dried and sputter-coated with gold (Cressington Sputter, Coater 108 auto, Watford, UK). Finally, the samples were examined with SEM Phillips XL-30 (Phillips, FEI, Hillsboro, OR, USA).

### 2.5. Biochemical Analysis of hUAs

Collagen quantification of native (*n* = 10, l = 2 cm) and decellularized (*n* = 10, l = 2 cm) hUAs was performed with a Hydroxyproline Assay Kit (MAK 009, Sigma-Aldrich, Darmstadt, Germany), according to the manufacturer’s instructions. Briefly, all samples were digested in 125 μg/mL papain buffer (Sigma-Aldrich, Darmstadt, Germany) at 60 °C for 12 h. Then, all samples were hydrolyzed with 12 M HCl, dried and incubated with Chloramine T/oxidation buffer and DMAB reagent. The hydroxyproline content, which corresponded to the collagen amount, was determined photometrically at 560 nm by interpolation to the hydroxyproline standard curve.

Accordingly, for the sGAG quantification, native (*n* = 10, l = 2 cm) and decellularized (*n* = 10, l = 2 cm) hUAs were digested in 125 μg/mL papain buffer. Then, in digested samples, the addition of dimethylene blue (Sigma-Aldrich, Darmstadt, Germany) was performed. Finally, the samples were quantified photometrically at 525 nm. The sGAG content was determined by interpolation to the standard curve (dilutions of 3, 6, 12, 25, 50, 100 and 150 μg/mL chondroitin sulfate were used for the development of the standard curve).

DNA quantification was performed in native (*n* = 10, l = 2 cm) and decellularized (*n* = 10, l = 2 cm) hUAs. To perform this quantification, all samples were digested in a lysis buffer that consisted of 0.1 M Tris pH 8, 0.2 M NaCl, 5 mM EDTA and 25 mg/mL Proteinase K (Sigma-Aldrich, Darmstadt, Germany), followed by incubation at 55 °C for 12 h. Inactivation of Proteinase K was performed after the complete tissue lysis, at 60 °C for 5 min. The DNA was isolated, cleaned and diluted in 50 μL of RNA-se-free water (Sigma-Aldrich, Darmstadt, Germany). Finally, the DNA amount in each sample was determined by photometrical measurement at 260 to 280 nm.

### 2.6. Preparation of hUAs for Biomechanical Analysis

Native (*n* = 10) and decellularized (*n* = 10) hUAs were analyzed for their biomechanical properties. Briefly, a ring-like (~1 mm width) and a strip-like sample (~5 mm long) were used for the qualitative histological observations. The hUAs (both native and decellularized) were cut into strips and ring samples along the circumferential and longitudinal direction, respectively. The occurred samples were placed in a Petri dish containing Krebs–Ringer solution at 37 °C. The frontal and the lateral aspects of the samples were observed under a stereomicroscope ((Nikon SMZ800; Nikon Instruments Europe BV, Amsterdam, The Netherlands) and images were acquired with a color digital camera (Leica DFC500, Leica Microsystems GmbH, Wetzlar, Germany). The inner and outer circumference, thickness, cross-sectional area and width of the ring and the strip samples were determined by measurements performed in the acquired images, using the Image-Pro Plus software (v 4.5, Media Cybernetics Inc., Bethesda, MD, USA).

### 2.7. Biomechanical Analysis of hUAs

For the biomechanical analysis of native (*n* = 10) and decellularized (*n* = 10) hUAs, an experimental device (Vitrodyne V1000 Universal Tester; Liveco Inc., Burlington, VT, USA) was used. The device consisted of (a) a stationary lower grip and an upper grip attached to the actuator, gradually extending the samples that were vertically mounted in the grips; (b) a load cell (GSO-250; Transducer Techniques, Temecula, CA, USA) with 0.01-g accuracy for the evaluation of load; (c) a rotary encoder providing feedback on the vertical displacement of the upper sample grip with 10-micrometer accuracy; (d) a saline bath wherein the samples were submerged during testing to sustain normal tissue hydration; (e) a heater coil (1130A, PolyScience, Niles, IL, USA) regulating the temperature of the saline bath at 37 °C; and (f) an accompanying personal computer, interfaced with the controller of the device via the Material Witness software package (v. 2.02, Liveco Inc., Burlington, VT, USA) to store the data. The ring and strip samples of hUAs were mounted in the device for the analysis, using hook-shaped grips. The unloaded length of all samples was obtained by vertically adjusting the upper grip to record only their weight. All samples were submitted to a progressively increasing tensile load at a 10 µm/s rate until full rupture of the wall.

### 2.8. Biomechanical Data Analysis

Stretch was calculated as the sample length during load increase by the experimental device (Vitrodyne V1000 Universal Tester; Liveco Inc., Burlington, VT, USA) divided by the unloaded sample length. The strain was calculated using the stretch values minus one. Stress was calculated by dividing the product of the load and stretch by their unloaded cross-sectional area, assuming tissue incompressibility. The stress–strain data were regressed with 9th-order polynomials, affording correlation coefficients r > 0.95, and the elastic modulus (tangent) at each strain level was calculated as the first derivative of stress over strain. Failure stress, representing tissue strength, and failure strain, representing extensibility, were calculated as the maximum stress and strain values at the first rupture. Peak elastic modulus, representing maximum tissue stiffness, was calculated as the highest elastic modulus value before the first rupture. All calculations were performed in Mathematica (v. 9.0, Wolfram Research Inc., Boston, MA, USA).

### 2.9. Quality Control of Isolated WJ-MSCs

MSCs used in this study were isolated from hUCs Wharton’s jelly tissue, as previously described [31,39]. The quality check of the WJ-MSCs (*n* = 5) at passage (P)1-P3 involved (a) the determination of morphological characteristics, (b) performance of tri-lineage differentiation, (c) colony-forming units’ (CFUs) assay performance and (d) immunophenotyping analysis using the flow cytometer.

The differentiation of WJ-MSCs into “osteocytes”, “adipocytes” and “chondrocytes” was achieved using the specific kits according to the manufacturer’s instructions. Specifically, the StemPro Osteogenesis, Adipogenesis and Chondrogenesis differentiation kits (Thermo Fischer Scientific, Waltham, MA, USA) were applied. To verify the differentiation efficiency of WJ-MSCs, histological stains were performed, as previously described [31,39]. For this purpose, Alizarin Red S, Oil Red O and Alcian Blue (Sigma-Aldrich, Darmstadt, Germany) were used for the determination of calcium deposition, lipid droplet and sulfated glycosaminoglycans’ (sGAGs) production, respectively.

CFUs assay was performed in WJ-MSCs at P1–P3. WJ-MSCs (from each passage) were detached from the culture flask using trypsin (0.025%)-EDTA (0.01%) buffer (Thermo Fischer Scientific, Waltham, MA, USA), counted, seeded at a density of 500 cells/well on 6-well plates and incubated for 15 days at 37 °C and 5% CO_2_. Then, the cultures were washed with PBS 1x (Sigma-Aldrich, Darmstadt, Germany) and formalin-fixed. Giemsa stain was applied for 5 min, and the stained CFUs were observed under an inverted Leica DM L2 light microscope (Leica, Microsystems, Weltzar, Germany). In addition, the positively stained CFUs were microscopically counted by two independent observers.

Flow cytometric analysis was performed in order to determine the WJ-MSCs’ immunophenotype, as has been proposed by ISCT [40]. For this purpose, 15 monoclonal antibodies (mAb) panel was used. This panel is composed of (a) fluorescein (FITC)-conjugated mAbs CD90, CD45, CD19, CD29, CD31 and HLA-ABC, (b) phycoerythrin (PE)-conjugated mAbs CD44, CD3, CD11b and CD34, (c) peridinin-chlorophyll-protein (PerCP)-conjugated mAbs CD105, HLA-DR and (d) allophycocyanin (APC)-conjugated mAbs CD73, CD10 and CD340. All mAbs were purchased from Becton Dickinson (BD biosciences, Franklin Lakes, NJ, USA). The immunophenotyping analysis was performed in FACS Calibur (BD biosciences, Franklin Lakes, NJ, USA) with at least 10,000 events at each tube. Flow cytometric data analysis was performed with FlowJo v10 (BD biosciences, NJ, USA).

For the below-described repopulation experiments, WJ-MSCs P3 were applied. In each passage, the total number and viability of WJ-MSCs were determined using automated count combined with trypan blue (Sigma-Aldrich, Darmstadt, Germany).

### 2.10. In Vitro Angiogenesis Assay

The ability of WJ-MSCs P3 to form networks was evaluated with the performance of an in vitro angiogenesis assay. Matrigel^©^ (BD, Heidelberg, Germany) was thawed on ice overnight, according to the manufacturer’s instructions. Then, 30 μL of the Matrigel^©^ was placed in each well of 24-well plate and incubated for 30 min at 37 °C. WJ-MSCs P3, at a density of 3 × 10^4^, were seeded into each well, followed by the addition of 500 μL of a-Minimum Essentials Medium (a-MEM) supplemented with Endothelial Growth Medium-2 (EGM-2). The networks were formed within 8 h. Images were acquired with a Leica DM L2 light microscope (Leica, Microsystems, Weltzar, Germany).

### 2.11. Repopulation of hUAs

For the repopulation experiments, Wharton’s Jelly (WJ)-MSCs (*n* = 5) of P3 were seeded onto the decellularized hUAs. Quality characteristics of WJ-MSCs including immunophenotyping analysis, trilineage differentiation, proliferation potential (until P3) and viability assessment, were performed, as described in the previous section (2.9 Quality Control of isolated WJ-MSCs).

To perform the repopulation experiments, decellularized hUAs (*n* = 10) were cut into rings (l = 1 cm) and were placed in 15 ml polypropylene conical falcon tubes. Then, MSCs at a density of 1.5 × 10^6^ cells were added to each hUA ring. Incubation at dynamic seeding conditions, using a thermal shaker, at 37 °C, for a maximum of 8 h, was performed. Then, the repopulated hUAs were placed into 6-well plates. The addition of WJ-MSCs P3 at a density of 1 × 10^5^ cells in the 6-well plates was also performed. Finally, the 6-well plates containing the repopulated hUAs were placed into the incubator at 37 °C and 5% CO_2_ for a time period of 30 days. Biweekly change of the culture media was performed to all repopulated hUAs. Repopulated hUAs were divided into the following two groups: group A—repopulated hUAs (*n* = 5) with WJ-MSCs P3 cultivated with regular culture medium consisted of α-MEM (Gibco, Thermo-Scientific, Waltham, MA, USA), 1% *v*/*v* Penicillin-Streptomycin (P-S, Gibco, Thermo-Scientific, Waltham, MA, USA), 1% *v*/*v* L-glutamine (L-glu, Gibco, Thermo-Scientific, Waltham, MA, USA) and 15% FBS (Gibco, Thermo-Scientific, Massachusetts, USA and group B—repopulated hUAs (*n* = 5) with WJ-MSCs P3 cultivated culture medium consisted of α-ΜΕΜ (Gibco, Thermo-Scientific, Waltham, MA, USA) supplemented with 1% P-S (Gibco, Thermo-Scientific, Waltham, MA, USA) and 15% *v*/*v* CBPL. The CBPL was produced based on a previously published protocol from our laboratory [30]. Briefly, for the production of CBPL, CBUs with an initial volume of 111–148 mL (including the anticoagulant) were used. None of the CBUs used for the production of CBPL met the minimum criteria of processing, cryopreservation and release outlined by the HCBB (Table S1). The CBUs were initially centrifuged at 210× *g* for 15 min at room temperature (RT). The top plasma fraction was transferred using a manual extraction system, to a secondary processing bag. The plasma fraction was centrifuged again at 2600× *g* for 15 min at RT. Finally, the supernatant platelet-poor plasma (PPP) was removed and the remaining CBPL (8–10 mL) was supplemented in α-ΜΕΜ. Additionally, a sample obtained from CBPL was taken and counted in a hematological analyzer (Sysmex XS 1000i, Roche, Basel, Switzerland) to verify the platelet concentration within the CBPL. The culture media were used from the initial WJ-MSCs seeding onto the decellularized hUAs.

### 2.12. Histological Analysis of the Repopulated hUAs

The evaluation of the repopulation efficiency of the decellularized hUAs was performed with the histological assessment. Briefly, repopulated hUAs (from both groups) were fixed in 10% *v*/*v* neutral formalin buffer, dehydrated, paraffin-embedded and sectioned at 5 μm, as previously described. H&E staining was performed for the evaluation of the seeded WJ-MSCs onto the hUAs.

The proliferation potential of WJ-MSCs P3 in seeded hUAs was assessed by indirect immunofluorescence experiments. The primary antibody used in this experimental procedure was anti-MAP kinase, activated dephosphorylated ERK 1 and 2 antibodies (1:1000, Sigma-Aldrich, Darmstadt, Germany), while the secondary was FITC-conjugated anti-mouse (1:80, Sigma-Aldrich, Darmstadt, Germany) antibody. Finally, the sections were dehydrated, and glycerol was mounted. The images were obtained with an LEICA SP5 II confocal microscope with LAS Suite v2 software (Leica Microsystems, GmbH, Wetzlar, Germany).

Quantification of MAP kinase expression and DAPI staining in repopulated hUAs based on immunofluorescence staining was performed as has been previously published by Prasad et al. [41]. For this purpose, the Image J (v1.533, National Institute of Health, USA) was used. Specifically, the acquired figures were converted into 8-bit images and then, using the Split Channels tool, were split into their original images. Plot profiles of MAP kinase expression (FITC, green channel) and DAPI (blue channel) were generated using the Histogram tool. The generated graphs represented the mean fluorescence intensities (MFI) corresponding to MAP kinase expression and DAPI stain.

### 2.13. Statistical Analysis

Graph Pad Prism v 6.01 (GraphPad Software, San Diego, CA, USA) was used for the statistical analysis in the current study. Comparisons of total hydroxyproline, sGAG and DNA contents and morphometric data between all samples were performed with Welch’s *t*-test. Comparison of DNA content and biomechanical results between all samples was performed with an unpaired non-parametric Kruskal–Wallis test. The statistically significant difference between group values was considered when *p*-value was less than 0.05. Indicated values were presented as mean ± standard deviation.

## 3. Results

### 3.1. Histological Analysis of hUAs

**Figure 1:**
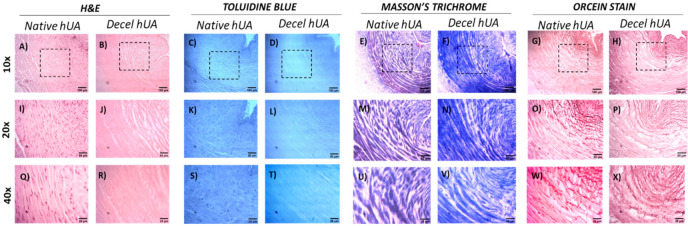
Histological analysis of hUAs (including native and decellularized samples). Native and decellularized hUAs stained with H&E (**A**,**I**,**Q** and **B**,**J**,**R**), TB (**C**,**K**,**S** and **D**,**L**,**T**), MT (**E**,**M**,**U** and **F**,**N**,**V**) and OS (**G**,**O**,**W** and **H**,**P**,**X**). The black boxes indicated the magnified field of 20× and 40× images. Images were presented with original magnification 10×, scale bars 100 μm, 20×, scale bars 50 μm and 40×, scale bars 25 μm. H&E: Hematoxylin and Eosin, TB: Toluidine Blue, MT: Masson’s Trichrome, OS: Orcein Stain.

**Figure 2:**
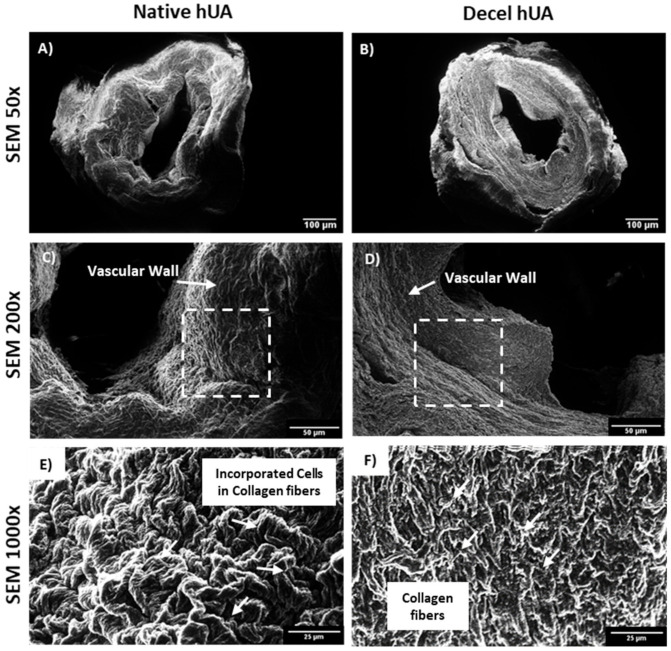
SEM histological analysis of hUAs. SEM images of native (**A**,**C**,**E**) and decellularized (**B**,**D**,**F**) hUAs. White arrows in images (**A**,**D**) represent the vascular wall. White squares in the same images represent the magnified region, as presented in images (**E**,**F**). White arrows in image E represent the combination of cells and collagen fibers of native, while in Figure **F**, white arrows represent only the preserved collagen fibers of decellularized hUAs. Images were presented with original magnification 50× (**A**,**B**), scale bars 100 μm, 200× (**C**,**D**), scale bars and 1000× (**E**,**F**), scale bars 25 μm.

The impact of the decellularization approach in the ECM of the hUAs was evaluated using histological analysis. In this way, H&E, TB, MT and OS stains were applied in the native and decellularized hUAs for the evaluation of cell presence, sGAGs, collagen and elastin, respectively. The results of H&E indicated the absence of cell and nuclear remnants in the decellularized hUAs, while the ECM was adequately preserved (Figure 1). On the other hand, regarding the sGAG, a weaker stain intensity was observed in the decellularized hUAs (Figure 1). The collagen and the entire ECM were preserved in UAs after the decellularization, as it was indicated by an MT stain (Figure 1). In addition, OS confirmed the presence of elastin both in the native and decellularized hUAs. The histological analysis revealed that the decellularized hUAs were characterized by a more compact structure, compared to the non-decellularized native hUAs. Further histological examination of the inner structure and morphology of the hUAs was conducted using SEM analysis (Figure 2). The decellularized hUAs were free from their cellular populations (endothelial cells and smooth muscle cells, Figure 2). Moreover, SEM analysis revealed the successful preservation of ECM structures, thus further confirming the initial histological analysis (involved H&E, AB and MT stains).

### 3.2. Biochemical Evaluation of hUAs

**Figure 3:**
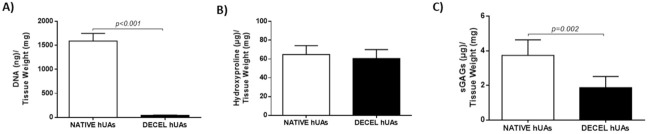
Biochemical analysis of hUAs. The biochemical analysis involved the DNA quantification (**A**), the hydroxyproline content (**B**) and sGAG content (**C**) quantification of native and decellularized hUAs. Statistically significant differences regarding the DNA (*p* < 0.001) and sGAG (*p* < 0.01) content were observed between native and decellularized hUAs.

In the current study, DNA, hydroxyproline and sGAGs were quantified in order to properly evaluate the impact of the decellularization procedure on hUAs. Specifically, DNA was eliminated in decellularized hUAs. Specifically, the DNA content of the native hUAs was 1589 ± 150 ng DNA/mg of tissue weight. In the decellularized hUAs, the DNA content was 43 ± 6 ng DNA/mg of tissue weight, suggesting that 97% of the initial DNA content was removed (Figure 3A and Table S2).

Regarding the hydroxyproline (which corresponds to collagen content) content, no statistically significant difference was observed between the native and decellularized hUAs. The hydroxyproline content of the native and decellularized hUAs was 65 ± 9 and 61 ± 9 μg hydroxyproline/mg of tissue weight, respectively (Figure 3B). Finally, the sGAG content in the native and decellularized hUAs was 4 ± 1 and 2 ± 1 μg sGAGs/mg of tissue weight, respectively (Figure 3C). Statistically significant differences were observed in the DNA content (*p* < 0.001) and the sGAG content (*p* < 0.01) between the native and decellularized hUAs.

### 3.3. Biomechanical Analysis of hUAs

**Figure 4:**
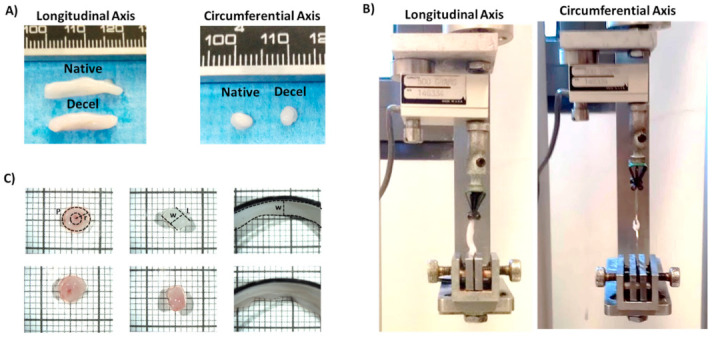
Set up of uniaxial biomechanical analysis of hUAs. Overview of decellularized hUAs in longitudinal and circumferential axis (**A**). Biomechanical testing of hUAs either in circumferential or longitudinal axis (**B**). Determination of hUAs dimensions using the stereoscope (**C**). P: Perimeter, r: radial, W: Width, L: Length.

**Figure 5:**
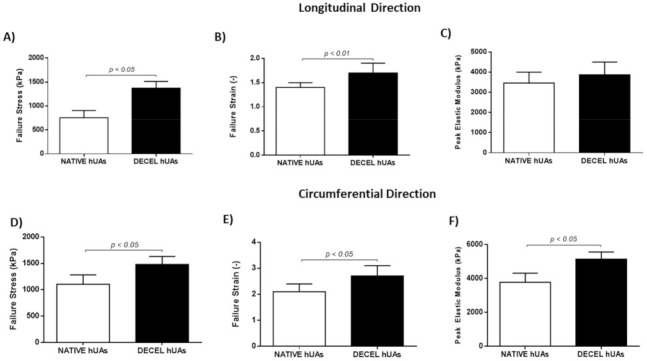
Biomechanical analysis of native and decellularized hUAs. Native and decellularized hUAs were tested for the maximum strain (**A**,**D**), failure strain (**B**,**E**) and peak elastic modulus (**C**,**F**), in the longitudinal (**A**–**C**) and circumferential (**D**–**F**) direction, respectively. Statistically significant differences between native and decellularized hUAs were found in failure stress (*p* < 0.05, in both directions), peak elastic modulus (*p* < 0.05, circumferential direction) and failure strain (*p* < 0.01 for the longitudinal direction and *p* < 0.05 for the circumferential direction). DECEL: Decellularized.

The biomechanical properties of the native and decellularized hUAs were determined with the performance of uniaxial testing. In this way, both the native and decellularized hUAs were tested in longitudinal and circumferential directions. Regarding the longitudinal direction, the failure stress (σ), failure strain and peak elastic modulus for the native and decellularized hUAs were 755 ± 150 and 1373 ± 140 kPa, 1.4 ± 0.1 and 1.7 ± 0.2, 3458 ± 548 and 3867 ± 630 kPa, respectively (Figure 4 and Figure 5A–C and Table S3). In the circumferential direction, the failure stress (σ), failure strain and peak elastic modulus for the native and decellularized hUAs were 1102 ± 180 and 1480 ± 150 kPa, 2.1 ± 0.3 and 2.7 ± 0.4, 3781 ± 540 and 5153 ± 420 kPa, respectively (Figure 4 and Figure 5D–F and Table S3). Statistically significant differences regarding the longitudinal direction between the native and decellularized hUAs were observed in failure stress (*p* < 0.05) and strain (*p* < 0.01, Figure 5A–C). Regarding the circumferential direction, statistically significant differences between the native and decellularized hUAs were observed in failure stress (*p* < 0.05), failure strain (*p* < 0.05) and peak elastic modulus (*p* < 0.05, Figure 5D–F).

### 3.4. WJ-MSCs Characterization

In this study, the WJ-MSCs P3 were applied for the in vitro recellularization of the decellularized hUAs. However, before further processing, the verification of the stem cell characteristics of the WJ-MSCs was performed.

**Figure 6:**
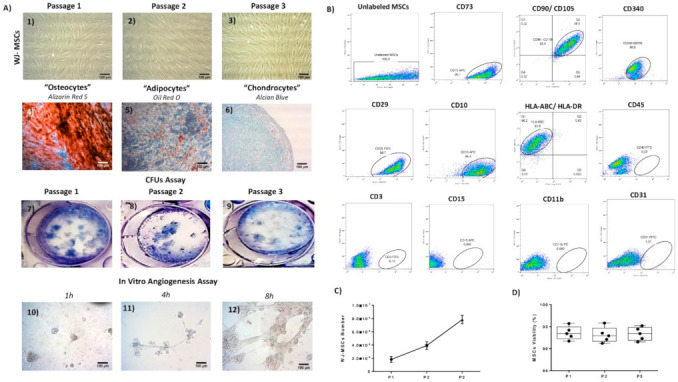
Characterization of the isolated WJ-MSCs. Morphological characteristics of WJ-MSCs (**A1**–**A3**). Differentiation of WJ-MSCs towards “osteogenic”, “adipogenic” and “chondrogenic” lineages (**A4**–**A6)**. The successful differentiation of WJ-MSCs into “osteocytes”, “adipocytes” and “chondrocytes” was verified using the histological stains Alizarin Red S, Oil Red O and Alcian Blue, respectively. CFUs assay of WJ-MSCs at P1 to P3 (**A7**–**A9**). In vitro angiogenesis assay performance. Images showing the developed network were acquired after 1, 4 and 8 h (**A10**–**A12**). Immunophenotyping analysis of WJ-MSCs P3 (**B**). Determination of total number (**C**) and viability of WJ-MSCs at P1–P3 (**D**). The images **A1**–**A6** and **A10**–**A12** were acquired with original magnification 10×, and scale bars 100 μm.

The WJ-MSCs at P1–P3 presented a fibroblastic-like morphology. No change in their morphological features was observed between the passages (Figure 6A). The WJ-MSCs P3 differentiated successfully to “osteogenic”, “adipogenic” and “chondrogenic” lineages upon stimulation with specific differentiation media. To determine the efficacy of the differentiation, histological stains were applied. Specifically, the mineral production (Ca^2+^ and Mg^2+^) from the differentiated WJ-MSCs were determined with the performance of Alizarin Red S. Indeed, a high number of calcium deposits to the differentiated “osteocytes” were observed (Figure 6A). Moreover, successful CFUs development was performed by the WJ-MSCs from P1–P3 (Figure 6A). The corresponded CFU numbers developed by the WJ-MSCs at P1, P2 and P3 were 12.3 ± 1.6, 12.1 ± 1.5 and 13.1 ± 1, respectively. No statistically significant differences regarding the CFU number were observed between the different WJ-MSCs passages (Figure S1). Additionally, to verify the WJ-MSCs’ properties to form an organized network, an angiogenesis assay was applied (Figure 6A). The WJ-MSCs started to develop the tubular networks after 4 h. An organized tubular network, formed by WJ-MSCs, was observed after 8 h of incubation.

The immunophenotyping analysis of the WJ-MSCs P3 showed an expression >90% for the CD73, CD90, CD105, CD29, CD10, CD44, CD340 and HLA-ABC expression <2% for the CD3, CD19, CD34, CD45, CD15, CD11b, CD31 and HLA-DR (Figure 6B, Figure S2 and Table S4). Finally, the WJ-MSCs were expanded successfully since their first isolation until reached P3 (Figure 6C). Specifically, the mean number of WJ-MSCs at P1 was 1.8 × 10^6^, at P2 it was 3.9 × 10^6^ and at P3 it was 7.8 × 10^6^ (Figure 6C). The viability of the WJ-MSCs at P1, P2 and P3 was 93.6 ± 1.3%, 93.1 ± 1.5% and 93.3 ± 1.3%, respectively, as confirmed by the trypan blue assay (Figure 6D).

### 3.5. Recellularization of hUAs

**Figure 7:**
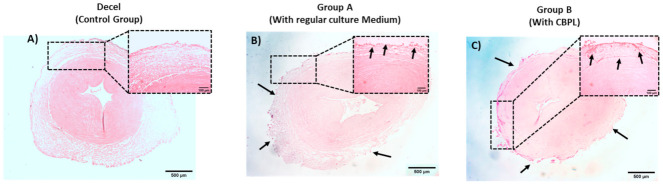
Histological analysis of hUAs (**A**–**C**). Overview of repopulated hUAs of groups A and B (**B**,**C**). Decellularized hUA served as the control group (**A**). Repopulated hUA of group A (**B**). WJ-MSCs P3 in group A were located only to the tunica adventitia. Repopulated hUA of group B (**C**). On the contrary, WJ-MSCs P3 in group B migrated successfully to the inner vascular wall. Images represented with original magnification 2.5× and scale bars 500 μm. Images in the black squares represented with original magnification 10×, scale bars 100 μm.

**Figure 8:**
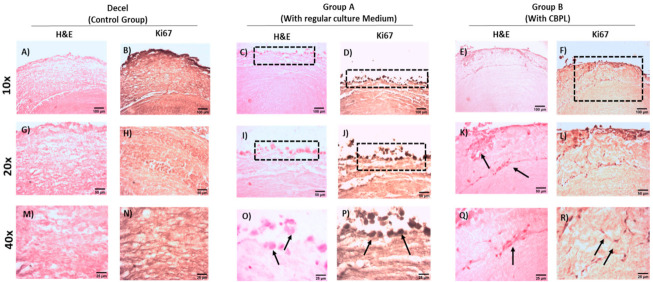
Histological analysis of repopulated vascular grafts with WJ-MSCs P3, located in the tunica adventitia. Decellularized hUAs stained with H&E (**A**,**G**,**M**). Repopulated hUAs of group A (**C**,**I**,**O**) and group B (**E**,**K**,**Q**) stained with H&E. Immunohistochemistry against Ki67 in decellularized hUAs (**B**,**H**,**N**), and repopulated hUAs of group A (**D**,**J**,**P**) and group B (**F**,**L**,**R**). Images (**A**–**F**) presented with original magnification 10×, scale bars 100 μm. Images (**G**–**L**) presented with original magnification 20×, scale bars 50 μm. Images (**M**–**R**) presented with original magnification 40×, scale bars 25 μm.

The WJ-MSCs P3 successfully repopulated the decellularized hUAs in both groups. However, a more uniform repopulation of the hUAs was observed when CBPL was used (Figure 7) Indeed, when CBPL was used as a supplement of the culture medium, a better distribution of the WJ-MSCs P3 was observed, compared to group A. H&E stain confirmed the presence of the WJ-MSCs P3 to the TA of hUAs in both groups (Figure 8). However, after 30 days of incubation, an extensive migration of cells from TA to TI was reported only in group B (Figure 8). The WJ-MSCs P3 of group A did not migrate, thus they were located only in the TA of the hUAs. Moreover, a greater number of the WJ-MSCs P3 were observed in group B compared to group A.

**Figure 9:**
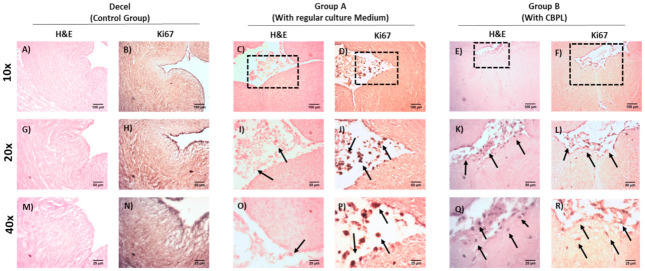
Histological analysis of repopulated vascular grafts with WJ-MSCs P3, located in the tunica intima. Decellularized hUAs stained with H&E (**A**,**G**,**M**). Repopulated hUAs of group A (**C**,**I**,**O**) and group B (**E**,**K**,**Q**) stained with H&E. Immunohistochemistry against Ki67 in decellularized hUAs (**B**,**H**,**N**), and repopulated hUAs of group A (**D**,**J**,**P**) and group B (**F**,**L**,**R**). Images (**A**–**F**) presented with original magnification 10×, scale bars 100 μm. Images (**G**–**L**) presented with original magnification 20×, scale bars 50 μm. Images (**M**–**R**) presented with original magnification 40×, scale bars 25 μm.

To further confirm the proliferative activity of the WJ-MSCs P3 in the repopulated hUAs, immunohistochemistry against Ki67 was performed (Figure 9). The expression of Ki67 was confirmed in both groups. However, a greater distribution of Ki67 was observed in group B, further confirming the H&E staining results.

**Figure 10:**
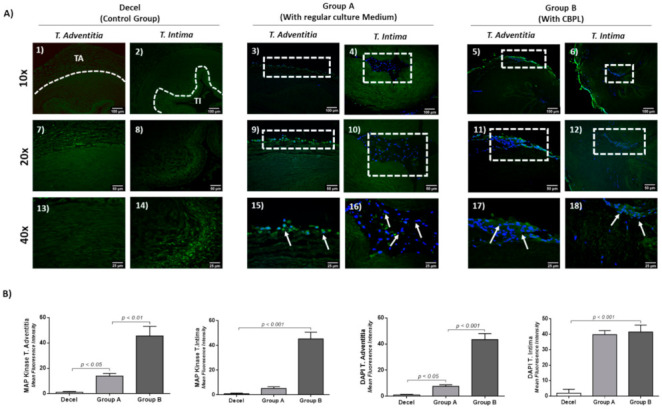
Indirect immunofluorescence against MAP kinase in combination with DAPI stain in repopulated hUAs (**A**). Decellularized hUAs did not exhibit any expression of anti-MAP kinase or DAPI stain (**1**,**2**,**7**,**8**,**13**,**14**) either in tunica adventitia or tunica intima regions. Repopulated hUAs in group A (cultured with regular medium) were characterized by both anti-MAP expression and DAPI stain (**3**,**9**,**15**,**4**,**10**,**16**). However, both signals were restricted only to the tunica adventitia of the repopulated hUAs (**3**,**9**,**15**). Repopulated hUAs in group B (with the use of CBPL) positively expressed the MAP kinase and DAPI stain both in tunica adventitia and tunica intima regions (**5**,**11**,**17**,**6**,**12**,**18**). Images (**1**–**6**) presented with original magnification 10×, scale bars 100 μm. Images (**7**–**12**) presented with original magnification 20×, and scale bars 50 μm. Images (**13**–**18**) presented with original magnification 40×, and scale bars 25 μm. Mean Fluorescence Intensity of MAP kinase and DAPI stain (**B**). Statistically significant differences regarding the MAP kinase expression and DAPI stain both in tunica adventitia (*p* < 0.01) and tunica intima (*p* < 0.001) in all groups. TA: Tunica Adventitia, TI: Tunica Intima. White boxes and arrows presented the presence of cells in repopulated hUAs.

The immunofluorescence results indicated the expression of MAP kinase in both groups (Figure 10 and Figure S3). However, the greater distribution and expression of MAP kinase were observed in group B in comparison to group A (Figure 10). The MFI of the MAP kinase expression in TA and TI in repopulated hUAs of group A and group B was 13.9 ± 2.1 and 1.1 ± 0.3, and 45.6 ± 7.1 and 45.3 ± 5.1, respectively. Accordingly, for the DAPI stain, the MFI in repopulated hUAs of groups A and B were 7.6 ± 1.1 and 39.8 ± 2.5, and 43.4 ± 4.4 and 41.5 ± 4.3, respectively. The statistically significant differences were observed in the study groups, regarding either the MAP kinase expression (*p* < 0.01) or the DAPI stain intensity (*p* < 0.001). The latter further confirms the greater proliferative potential and migratory ability of the WJ-MSCs P3 in decellularized hUAs, when CBPL was also used as a supplement in the culture medium.

## 4. Discussion

The fabrication of functional bioengineered SDVGs, suitable for CVD surgery, represents one of the major challenges of blood vessel engineering [15]. Current knowledge from the already performed research has shown that acellular SDVGs cannot be applied in patients due to severe host adverse reactions, such as thrombus and neointima formation [42,43]. In addition, decellularized animal vessels, cross-linked, sterilized, cryopreserved allografts or commercially available SDVGs fail to integrate properly to the damaged region [44,45,46,47,48,49,50,51,52,53,54,55,56,57,58]. Consequently, the host inflammatory response attributed by neutrophils and M1 macrophages is initiated, leading to platelet activation and aggregation [59]. Additionally, the cryopreserved allografts are characterized by increased bacterial infections [60]. In this way, the development of well-defined SDVGs requires further evaluation.

For this purpose, the repopulation of the decellularized SDVGs with host cellular populations may attenuate the aforementioned lethal consequences. The proper repopulation of the decellularized vascular grafts can be performed with the use of a suitable bioreactor system [61]. In this process, the optimum conditions for the repopulation of the grafts can be adjusted, ensuring the uniform distribution and proliferation of the cellular populations [62]. However, the whole process requires further improvement in order to reduce the fabrication time of the vascular graft.

In the majority of the studies, culture media utilizing FBS and synthetic growth factors are mostly applied [62,63,64]. FBS is a rich source of growth factors and hormones, which is commonly used as a culture media additive for the in vitro isolation and expansion of cells [65,66,67,68]. On the other hand, it has been shown, that significant variation in FBS content may exist between different lots [65,66,67,68]. Additionally, FBS may contain prions, xenogeneic antigens and bovine proteins, which can cause allergic reactions or the transmission of zoonotic diseases to the host [65,66,67,68].

Therefore, the utilization of better-defined supplements for the repopulation and fabrication of bioengineered SDVGs is an important asset. Previously conducted studies have shown that peripheral blood (PB) or cord blood derivatives, such as platelet-rich plasma (PRP) or platelet lysate (PL), may sustain the stem cell proliferation and, thus, can be used as an alternative to FBS supplement in the culture media [32,66,67,68,69]. Either PBPL or CBPL can induce the expansion of MSCs in great numbers, without altering their stemness properties [32,69]. CBPL has previously been used in combination with ascorbic acid for the development of vascular smooth muscle cells originating from MSCs [70].

The current study aimed to provide insight evidence regarding the beneficial use of CBPL in the repopulation of the decellularized SDVGs. For this purpose, the hUAs were decellularized effectively and served as scaffolds. Furthermore, WJ-MSCs were used as the cell population for the repopulation assays. As it has been shown previously by our group, hUAs can be efficiently decellularized, serving as an ideal scaffold for cell repopulation [39]. The preservation of the key specific ECM proteins in decellularized matrices, is of major importance, promoting the development of a suitable microenvironment for cell infiltration. In our study, the preservation of the ECM proteins was confirmed by the performance of the histological analysis. An H&E stain initially confirmed the preservation of an hUA ultrastructure, while at the same time no cell or nuclei materials were evident in decellularized vessels. Besides that, a more comprehensive analysis of hUAs’ ECM involved the performance of TB, MT and OS. The above histological stains can specifically detect the sGAGs, collagen and elastin in the vessel wall of the hUAs. Indeed, MT and ES revealed the presence of collagen and elastin in the decellularized vessels in a similar way to the native ones. On the contrary, a weaker TB stain was observed in the decellularized hUAs, compared to the native hUAs, reflecting the possible reduction in the sGAG content. Moreover, the decellularized hUAs retained their initial collagen and elastin alignment to the vascular walls. This important finding has been related to improved biomechanical properties and better cell infiltration. The properly aligned collagen and elastin fibers retain their initial adhesion positions, which are important for cell infiltration, proliferation and differentiation. Indeed, these processes are mainly attributed by interactions between cell integrins (α1β1, α2β1 and ανβ1) with the RGD binding motifs (arginine-glycine and aspartate), which are found in collagen and elastin fibers [71,72]. Similar results have been reported in the past by other research groups, thus further confirming the effective decellularization of the hUAs. Indeed, the successful preservation of fibronectin in decellularized hUAs, a protein that exerts important key-binding activities and has previously been shown by our research team [38,73]. Fibronectin, in a similar way as collagen and elastin, contains RGD binding motifs; therefore, mediated cell adhesion through integrins can be performed. On the other hand, decellularized SDVGs may need an additional pre-coating with heparin and VEGF in order to enhance the anti-coagulant properties and ECs’ adhesion. Dimitrievska et al. [74] proposed a novel method for advanced heparin-binding in decellularized vascular grafts. This method is reliant on the covalent linking of high-density heparin in decellularized vessels, utilizing the “alkyne-azide” clickable dendrons. Furthermore, the same group showed that immobilized heparin induced a significant reduction in platelet adhesion, whereas the repopulation of the vessel with ECs was efficient. Koobatian et al. [75] showed that the addition of VEGF to the heparin binding domain may improve the long-term patency of the vascular grafts. VEGF favors the ECs migration and adhesion; therefore, a more uniform endothelium could be developed in the inner layer of the vascular grafts. Gui et al. [21] was the first who reported the efficient decellularization of hUAs and explored their potential use as SDVG. In this study, hUAs were decellularized, retaining their ultrastructure orientation in the same way as it has been reported in the current technical note. No discrepancies regarding the histological results were observed between the two studies. SEM images of native and decellularized hUAs further confirmed the production of a properly defined vascular graft. All the layers of the vascular wall (TI, TM and TA) were well preserved. No signs of ECM extensive destruction were observed in the decellularized hUAs.

Biochemical analysis of the collagen, sGAG and DNA content of the native and decellularized hUAs was performed in order to evaluate the decellularization process. The DNA content was eliminated in the decellularized hUAs. Additionally, the sGAG content showed a statistically significant decrease in the decellularized hUAs, compared to the native. On the other hand, the collagen content was preserved both in the native and decellularized vessels. The biochemical analysis results were in accordance with the histological stain observations. Indeed, the weaker TB stain in the decellularized hUAs was positively related to the loss of the sGAG content. At the same time, the absence of cell and nuclei materials confirmed the low DNA content in the decellularized vessels.

Due to the existence of variations between the native and decellularized hUAs, regarding the sGAG content and cell elimination, these may be related to the altered biomechanical properties of the decellularized hUAs. For this purpose, uniaxial biomechanical testing in longitudinal and circumferential directions was performed. Biomechanical differences were observed between the native and decellularized hUAs. These differences reflected the adaptation of a stronger and more extensible behavior in the decellularized hUAs compared to the native ones. Mostly, these differences existed only in the circumferential and not in the longitudinal direction. This alteration in biomechanical properties may be explained partially due to cell elimination and fiber disorganization. However, our histological analysis did not reveal collagen or elastin disorganization in the vascular wall of the decellularized hUAs. In addition, biochemical analysis revealed that decellularized hUAs were characterized by less sGAG content. Such alterations in the sGAG content, in combination with the loss of VSMCs, may cause the crimp of the collagen and elastin fibers. Τhis, in turn, may increase the crosslink between collagen and elastin fibers, thus explaining the stiffer behavior of the decellularized hUAs. The presented biomechanical results were in accordance with previously conducted studies [76,77]. This may suggest that the decellularization may have an impact on the properties of the decellularized vessels.

Once the properties of the decellularized vessels were established, an evaluation of the repopulation efficacy with the WJ-MSCs with or without the CBPL was performed. Before the repopulation assessment, the WJ-MSCs were isolated, expanded and their characteristics were determined based on the ISCT criteria [40]. WJ-MSCs compromise a fetal stem cell population with unique immunomodulatory and regenerative properties [77,78,79]. It is universally known that MSCs are lacking the expression of HLA class II molecules, and co-stimulatory molecules (such as CD40, CD80 and CD86) [78,79,80]. Therefore, MSCs can be universally applied in the allogeneic setting of the tissue engineering approaches. Moreover, fetal MSCs (such as the WJ-MSCs) are characterized by longer telomeres and increased telomerase activity, and at the same time, by no mutations or epigenetic modifications to their genome, compared to the adult MSCs (such as the bone marrow or adipode Tissue MSCs) [78,79,80,81,82,83]. In addition, fetal stem cells can be greater expanded in vitro without any chromosomal instability [81,82,83]. Therefore, fetal MSCs may be a greater source of stem cells compared to adult cells for regenerative medicine applications. In the current study, it was shown that WJ-MSCs are capable of contributing to a vascular network formation, reflecting their ability to differentiate to other cells such as the ECs [78,79,80,81,82,83]. In the context of tissue engineering, a great number of easily handled cells is required for the successful repopulation of the decellularized matrices. In this way, the WJ-MSCs could represent a desired stem cell source for the successful production of functional SDVGs.

In the context of the repopulation procedure, WJ-MSCs P3 (with or without the addition of CBPL) were dynamically seeded to the decellularized hUAs for a time period of 30 days. Then, a histological assessment was performed. In both groups, WJ-MSCs P3 were successfully seeded in the decellularized vessels. However, the WJ-MSCs of group A were restricted only to the outer layer of the vessel. On the other hand, in group B, the WJ-MSCs were distributed more uniformly compared to group A. In group B, the WJ-MSCs were observed to migrate toward the TM of the vessel. Furthermore, the WJ-MSCs in both groups showed positive expression for Ki67.

To date, the PL mostly derived from the peripheral blood has shown promising results, regarding cell proliferation and differentiation in 2D conditions. Doucet et al. [84] indicated the better proliferation and expansion potential of MSCs cultured with PL compared to those cultured with fetal calf serum (FCS). Jooybar et al. [85] showed that PL can be used in various tissue engineering approaches. Specifically, Jooybar et al. [85] developed a novel injectable platelet lysate-hyaluronic acid hydrogel. Then, bone marrow MSCs were encapsulated in the aforementioned hydrogel. The results of this study indicated the high expression of *AGGRECAN (ACAN)*, *COLLAGEN I*/*II* and *SRY-BOX TRANSCRIPTION FACTOR 9 (SOX9)*. The encapsulated MSCs presented an increased metabolic activity and differentiation potential towards chondrocytes. Zhang et al. [86] showed the beneficial properties of PL regarding the neo-vascularization potential of decellularized rat pancreatic scaffolds. Indeed, the contained growth factors of PL supported the ECs’ adhesion onto the decellularized pancreatic scaffolds. Finally, the repopulated scaffolds were implanted into the animal model. The results of this study showed that the released growth factors by the PL contribute significantly to better EC adhesion and vascular network development. The above pancreatic scaffolds were characterized by good biocompatibility, supporting, in this way, the long-term survival of the graft. However, until now, the CBPL has not been broadly applied in tissue engineering approaches. The current study represents a novel study, where the CBPL (with its contained growth factors) induced higher repopulation efficacy in decellularized hUAs, compared to the regular culture medium. In this way, CBPL may represent a better supplement for tissue engineering approaches, such as the production of functional bioengineered SDVGs.

Additional analysis of the repopulated vessels involved the detection of the dephosphorylated MAP kinase, using the immunofluorescence assay. Indeed, the WJ-MSCs in group B were expressed greater in the dephosphorylated MAP kinase, compared to group A. The monoclonal antibody (mAb), which was used, specifically recognized the activated MAP kinase isoforms ERK1 and ERK2. These isoforms are implicating in cell growth and proliferation [87,88]. It has been shown that ERK1/2 activation is required for the cells to move from the G0 to G1 phase, through the accumulation of phosphatidylinositol-3-OH kinase [89,90]. Accordingly, the overexpression of activated ERK1/2 in quiescent fibroblasts was sufficient to perform the S-phase entry [90]. Additionally, in loss of function experiments, using the PD98059 (an inhibitor of ERK1/2) observed a halt of the cell proliferation and growth factor production, which was acting with the tyrosine kinase receptors or G protein-coupled receptors in VSMCs [87,88]. Additionally, the use of another synthetic inhibitor (LY294002), specific for PI(3)K, blocked the DNA synthesis and the overall cell growth of cells [90,91,92,93]. This suggests that the activation of ERK1/2 plays a significant role in the downstream activation of other proteins such as PI(3)K, which contribute to cell growth and proliferation [90,91,92,93]. The activation of ERK1/2 can be promoted through the binding of various growth factors to their specific receptors. Among them, TGF-β1, VEGF, PDGF and FGF induce mitogenic benefits to cells through the activation of MAK isoforms [94,95,96,97]. Previous studies, conducted by our research team and also by others, have shown that CBPL is characterized by significant growth factor content [30,31]. TGF-β1, FGF, VEGF, PDGF, IGF, cytokines and chemokines are represented in the CBPL. In this way, it could be explained that repopulated vessels with CBPL were characterized by high WJ-MSCs proliferation and distribution to the vascular wall.

## 5. Conclusions

The current study described, in detail, the impact of the decellularization procedure to the vessels and the repopulation efficacy using the CBPL. CBPL, as a supplement to the culture media, may significantly improve the repopulation process. The future goal of our research will be the use of the CBPL culture medium in a vessel bioreactor system in order to assess if the proposed medium produced better-cellularized vessels. The utilization of CBPL may add more beneficial properties to the repopulated vessels, avoiding, in this way, any allergic reactions from the host. In turn, this may bring the production of fully personalized vascular grafts one step closer to clinical utility.

## Data Availability

Not applicable.

## Supplementary Materials

The following are available online at https://www.mdpi.com/article/10.3390/bioengineering8090118/s1. Figure S1. WJ-MSCs CFUs counting. No statistically significant differences were observed between WJ-MSCs at P1–P3. Figure S2. Flow Cytometric analysis of WJ-MSCs P3. High (A) and low (B) expression of CDs in WJ-MSCs P3. Figure S3. Indirect immunofluorescence against MAP kinase in combination with DAPI stain in repopulated hUAs. Indirect immunofluorescence of decel hUAs (A, B, G, H, M, N), group A (C, D, I, J, O, P) and group B (E, F, K, L, Q, R). Images A-R, presented with original magnification 40×, scale bars 25 μm. Table S1. Acceptable range of results outlined by the HCBB for processing and storage of cord blood units. Table S2. Raw data of DNA quantification. Table S3. Biomechanical analysis of native and decellularized hUAs. Statistically significant differences between the samples of longitudinal and circumferential direction were observed in failure stress (p< 0.01). Table S4. Flow Cytometric analysis of WJ-MSCs P3.
