# Supplementary material for: Improved Repopulation Efficacy of Decellularized Small Diameter Vascular Grafts Utilizing the Cord Blood Platelet Lysate
Source: Bioengineering (Basel). 2021 Aug 27;8(9):118. doi: 10.3390/bioengineering8090118 (PMC8467559; doi:10.3390/bioengineering8090118)
Supplement: Supplementary file 1 [file bioengineering-08-00118-s001.zip › bioengineering-1340186-supplementary.pdf]

Table S1. Acceptable range of results outlined by the HCBB for processing and storage of cord blood units.

Criteria	Unrelated Allogeneic	Related Allogeneic
**Collection bag mixed weight**	120 – 280 gr	100 – 220 gr
**WBC absolute count**	120 – 250 x 107 cells	100 – 250 x 107 cells
**WBC concentration**	13 – 29.5 cells/µL	10 – 29.5 cells/µL
**Cell Viability**	85 – 100%	85 – 100%

Table S2. Raw data of DNA quantification.

**DNA ng / mg Tissue Weight**		
**Sample No**	**Native hUAs**	**Decel hUAs**
1	1700	50
2	1350	45
3	1658	35
4	1750	40
5	1280	48
6	1710	35
7	1670	47
8	1642	50
9	1520	44
10	1610	37
Average	1589	43
StDev	150	6

Table S3. Biomechanical analysis of native and decellularized hUAs. Statistically significant differences between the samples of longitudinal and circumferential direction were observed in failure stress ($p<0.001$), failure strain ($p<0.001$) and peak elastic modulus ($p< 0.01$).

	**Longitudinal Direction**		**Circumferential Direction**		
	Native hUAs	***Decel hUAs***	***Native hUAs***	***Decel hUAs***	***p value***
Failure Stress (kPa)	755 ± 150	1373 ± 140	1102 ± 180	1480 ± 150	p< 0.001
Failure Strain (-)	1.4 ± 0.1	1.7 ± 0.2	2.1 ± 0.3	2.7 ± 0.4	p< 0.001
Peak Elastic Modulus (kPa)	3458 ± 540	3867 ± 630	3781 ± 540	5130 ± 420	p< 0.01

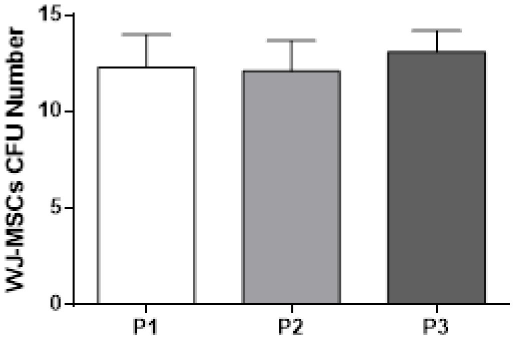

Figure S1. WJ-MSCs CFUs counting. No statistically significant differences were observed between WJ-MSCs at P1-P3.

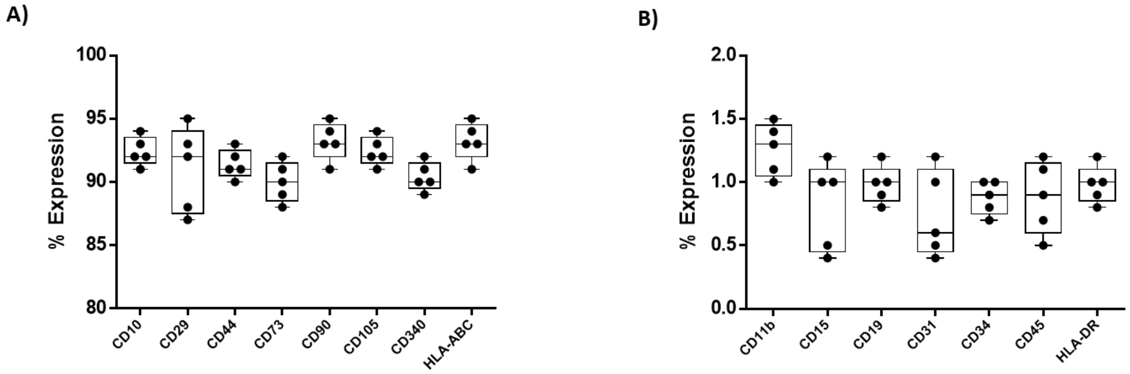

Figure S2. Flow Cytometric analysis of WJ-MSCs P3. High (A) and low (B) expression of CDs in WJ-MSCs P3.

Table S4. Flow Cytometric analysis of WJ-MSCs P3.

Abs	S1	S2	S3	S4	S5	Average	StDev
			High Expression				
CD10	91	93	94	92	92	92.4	1.0
CD29	88	95	87	92	93	91	3.0
CD44	91	92	90	93	91	91.4	1.0
CD73	88	89	91	90	92	90	1.4
CD90	91	93	95	94	93	93.2	1.3
CD105	91	92	93	94	92	92.4	1.0
CD340	90	89	90	91	92	90.4	1.0
HLA-ABC	91	93	94	95	93	93.2	1.3
			Low Expression				
CD11b	1.1	1.3	1.4	1	1.5	1.26	0.2
CD15	0.5	0.4	1	1.2	1	0.82	0.3
CD19	1	1.2	0.8	0.9	1	0.98	0.1
CD31	0.5	0.4	0.6	1.2	1	0.74	0.3
CD34	0.7	0.8	0.9	1	1	0.88	0.1
CD45	0.5	0.7	0.9	1.1	1.2	0.88	0.3
HLA-DR	1	1.2	0.8	0.9	1	0.98	0.1

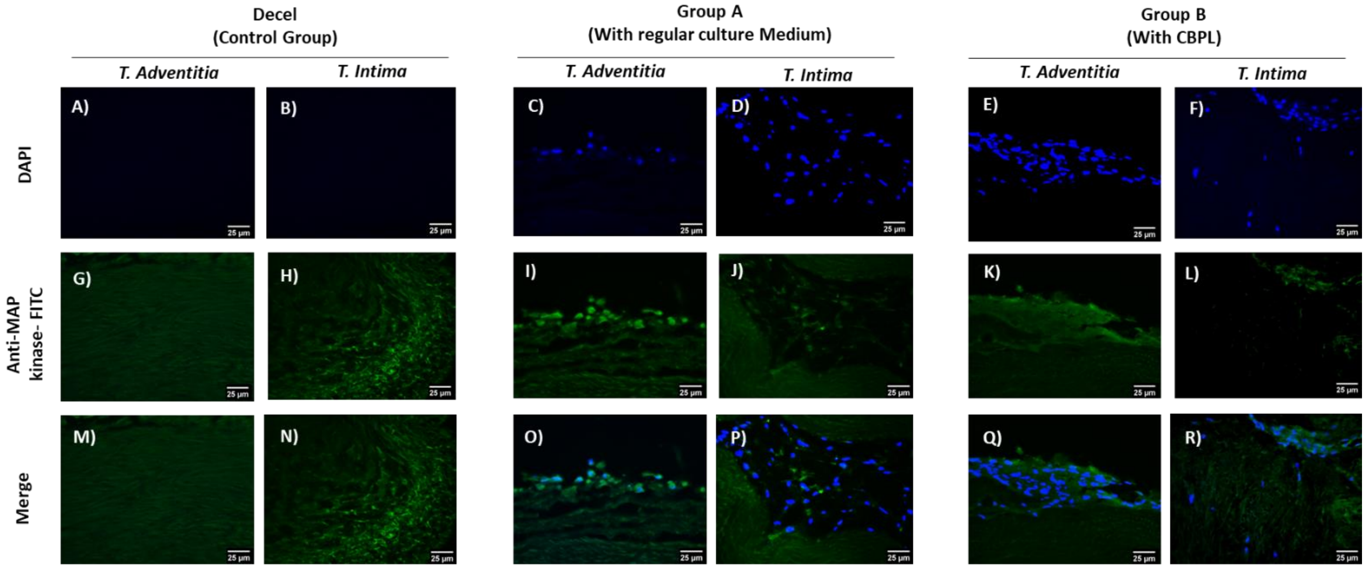

Figure S3. Indirect immunofluorescence against MAP kinase in combination with DAPI stain in repopulated hUAs. Indirect immunofluorescence of decel hUAs (A, B, G, H, M, N), group A (C, D, I, J, O, P) and group B (E, F, K, L, Q, R). Images A-R, presented with original magnification 40x, scale bars 25 µm.
